# Towards 90-90: Findings after two years of the HPTN 071 (PopART) cluster-randomized trial of a universal testing-and-treatment intervention in Zambia

**DOI:** 10.1371/journal.pone.0197904

**Published:** 2018-08-10

**Authors:** Sian Floyd, Helen Ayles, Albertus Schaap, Kwame Shanaube, David MacLeod, Mwelwa Phiri, Sam Griffith, Peter Bock, Nulda Beyers, Sarah Fidler, Richard Hayes, for the HPTN 071 (PopART) Study Team

**Affiliations:** 1 Department of Infectious Disease Epidemiology, London School of Hygiene & Tropical Medicine, London, United Kingdom; 2 Department of Clinical Research, London School of Hygiene & Tropical Medicine, London, United Kingdom; 3 Zambart, University of Zambia School of Medicine, Lusaka, Zambia; 4 FHI 360, HIV Prevention Trials Network, Durham, North Carolina, United States of America; 5 Desmond Tutu TB Centre, Department of Paediatrics and Child Health, Stellenbosch University, Stellenbosch, South Africa; 6 HIV Clinical Trials Unit, Imperial College London, London, United Kingdom; University of California, Berkeley, UNITED STATES

## Abstract

**Background:**

HPTN071(PopART) is a 3-arm community-randomised study in 21 peri-urban/urban communities in Zambia and the Western Cape of South Africa, with high HIV prevalence and high mobility especially among young adults. In Arm A communities, from November 2013 community HIV care providers (CHiPs) have delivered the “PopART” universal-test-and-treat (UTT) package in annual rounds, during which they visit all households and offer HIV testing. CHiPs refer HIV-positive (HIV+) individuals to routine HIV clinic services, where universal ART (irrespective of CD4 count) is offered, with re-visits to support linkage to care. The overall goal is to reduce population-level adult HIV incidence, through achieving high HIV testing and treatment coverage.

**Methods and findings:**

The second annual round was June 2015-October 2016. Included in analysis are all individuals aged ≥15 years who consented to participate, with extrapolation to the total population. Our three main outcomes are (1) knowledge of HIV+ status (2) ART coverage, by the end of Round 2 (R2) and compared with the start of R2, and (3) retention on ART on the day of consenting to participate in R2. We used “time-to-event” methods to estimate the median time to start ART after referral to care. CHiPs visited 45,631 households during R2, ~98% of the estimated total across the four communities, and for 94% (43,022/45,631) consent was given for all household members to be listed on the CHiPs’ electronic register; 120,272 individuals aged ≥15 years were listed, among whom 64% of men (37,265/57,901) and 86% (53,516/62,371) of women consented to participate in R2. We estimated there were 6,521 HIV+ men and 10,690 HIV+ women in the total population of visited households; and that ~80% and ~90% of HIV+ men and women respectively knew their HIV+ status by the end of R2, fairly similar across age groups but lower among those who did not participate in Round 1 (R1). Among those who knew their HIV+ status, ~80% of both men and women were on ART by the end of R2, close to 90% among men aged ≥45 and women aged ≥35 years, but lower among younger adults, those who were resident in R1 but did not participate in R1, and those who were newly resident in the area of the community in which they were living in R2. Overall ART coverage was ~65% among HIV+ men and ~75% among HIV+ women, compared with the cumulative 90–90 target of 81%. Among those who reported ever taking ART, 93% of men and 95% of women self-reported they were on ART and missed 0 pills in the last 3 days. The median time to start ART after referral to care was ~6 months in R2, similar across the age range 25–54 years, compared with ~9.5 months in R1. The two main limitations to our findings were that a comparison with control-arm communities cannot be made until the end of the study; and that to extrapolate to the total population, assumptions were required about individuals who were resident, but did not participate, in R2.

**Conclusions:**

Overall coverage against the 90–90 targets was high after two years of intervention, but was lower among men, individuals aged 18–34 years, and those who did not participate in R1. Our findings reflect the relative difficulties for CHiPs to contact men at home, compared with women, and that it is challenging to reach high levels of testing and treatment coverage in communities with substantial mobility and in-migration. The shortened time to start ART after referral to care in R2, compared with R1, was likely attributable to multiple factors including an increased focus of the CHiPs on linkage to care; increasing community acceptance and understanding of the CHiPs, and of ART and UTT, with time; increased coordination with the clinics to facilitate linkage; and clinic improvements.

## Introduction

The UNAIDS 90-90-90 targets, set for 2020, are that 90% of HIV-positive individuals know their HIV-positive status, 90% of those who know their HIV-positive status are on antiretroviral therapy (ART), and 90% of those taking ART are virally suppressed[1]. These targets correspond to 81% of HIV-positive individuals being on ART and 73% of HIV-positive individuals being virally suppressed, with the overall goal of improving the health of people living with HIV and decreasing HIV transmission at the population level.

Evidence that universal ART, irrespective of CD4 count, would contribute to improving the health and survival of people living with HIV became available from two randomized controlled trials in 2015[2, 3]. Modelling, drawing on the findings of the HPTN052 trial, has shown that reaching 90-90-90 has the *potential* to decrease transmission at population level[4–6], although this remains to be proven in a rigorously designed trial. Empirical findings from two population-intervention platforms have also indicated that higher ART coverage levels are associated with lower HIV incidence in the general adult population, through a comparison across time periods (Rakai district, Uganda) or across areas (rural KwaZulu Natal, South Africa)[7, 8].

In contrast, the community-randomised ANRS 12249 TasP trial of universal-testing-and-treatment (UTT), conducted in rural KwaZulu Natal in South Africa, found no evidence of an effect of the intervention on HIV incidence[9, 10]. However, the percentage of HIV-positive adults who were on ART at the end of this trial was similar in the two study arms, and much lower than the cumulative 90–90 target of 81%[10], so the trial findings do not refute the hypothesis that UTT can reduce HIV transmission. Three other community-randomised trials (CRTs) to evaluate the effect of UTT interventions on population-level adult HIV incidence have been underway since 2013–14, and will complete in 2017–19[11–13]. They will provide evidence from rural settings in Uganda and Kenya[13], rural and peri-urban settings in Botswana[12], and peri-urban and urban settings in Zambia and the Western Cape of South Africa[11].

During 2012–2014, most countries in sub-Saharan Africa were far from reaching the 90-90-90 targets[14, 15]. Whilst WHO guidelines were updated in 2015 to recommend universal ART[16], this might not be sufficient to reach the 90-90-90 targets; innovations to increase the uptake of HIV testing, linkage to HIV care following an HIV-positive diagnosis, the rapidity of ART initiation following linkage to care, and treatment adherence, may also be required. In 2016, “Population-based HIV Impact Assessment” (PHIA) surveys found that in Malawi ~70%, and in Zambia and Zimbabwe ~60%, of all HIV-positive adults (regardless of their self-reported knowledge of their HIV-positive status and use of ART) were virally suppressed, showing that the way in which these countries have scaled up testing, treatment, and treatment monitoring and adherence support, services, has enabled them to approach the 90-90-90 targets[17]. Additionally, a survey conducted in 2013–2015 in Botswana, as part of the Botswana-Combination-Prevention-Project (BCPP) CRT, found that ~70% of all HIV-positive adults were virally suppressed prior to the roll-out of study interventions[12]. The findings from these four settings indicate that, with appropriate interventions, it may be possible to reach the cumulative 90-90-90 target.

The HPTN 071 (PopART) trial is a 3-arm CRT in 21 large peri-urban/urban communities in Zambia and the Western Cape of South Africa, with high HIV prevalence and high mobility especially among young adults, and a total study population of ~1 million people[11]. The study is testing the impact on HIV incidence of a household-based combination HIV prevention approach (Arms A and B) provided by community HIV care providers (CHiPs) compared with standard-of-care (Arm C). In Arm A and B communities, CHiPs have offered *universal testing* and support for linkage to HIV care and treatment adherence, while in Arm A (but not Arm B) *universal treatment* has been delivered through routine government services, from January 2014. During May/June 2016 in Zambia, and during October 2016 in South Africa, the clinics in all Arm B and Arm C communities transitioned to offer universal treatment.

Across the four Arm A Zambian communities, we have previously estimated that immediately prior to the first year of intervention ~44% of HIV-positive adults were on ART, and that this increased to ~61% by the end of the first year of intervention in mid-2015[18]. An important finding from year 1 was that ART coverage was lower among men than women, and lower among younger than older adults, consistent with other studies[12, 17]. In this paper, we report findings from these same four Zambia communities about how close we have come to the 90–90 targets by October 2016, after two years of delivering the PopART UTT intervention, disaggregated by gender and age group and including information on adolescents aged 15–17 years for the first time. We also disaggregate our findings according to whether or not an individual participated in year 1, because it became apparent during year 2 that there was high mobility within, and into and out of, the study communities. In addition, we present findings about whether the time to start ART after referral to HIV care by CHiPs was shortened in the second year of intervention compared with the first year.

## Methods

### Setting

The study population consists of the four Arm A communities in Zambia, across which adult HIV prevalence ranges from ~10% to ~25% and the total population is ~200,000[11].

### PopART intervention

In Arm A, CHiPs deliver the PopART “UTT” intervention. This consists of offering universal home-based HIV testing, referral of HIV-positive (HIV+) individuals to routine government HIV clinic services that offer universal ART, and re-visits to HIV-positive individuals to support linkage to HIV care and retention on ART. The PopART intervention also includes various other services—HIV education, referral to medical male circumcision services, referral to antenatal care for pregnant women, reproductive health and family planning referrals, tuberculosis symptom screening followed by referral of those with symptoms to the clinic for diagnosis, sexually-transmitted-infection symptom screening followed by referral of those with symptoms to the clinic for diagnosis, and condom provision—providing a combination HIV prevention package[18].

Before the start of the PopART intervention, each of the intervention communities were divided into “zones” with on average ~500 households. CHiPs work in pairs, with each team responsible for one zone. CHiPs deliver the “PopART” intervention in annual “rounds”, during which they visit all households (irrespective of whether or not a household was resident during a previous round), offer to explain the intervention, and ask permission to enumerate (list) all household members. The first “annual round” (Round 1) was from November 2013 to June 2015, and the second “annual round” (Round 2) was from June 2015 to October 2016, with the intervention rolled out across communities over a 1–2 month period, and within communities across all zones simultaneously.

In Round 1 (R1) and Round 2 (R2), CHiPs aimed to contact all adults (≥18 years) at least once, and from early in Round 2 (October 1 2015) this was also the aim for those aged 15–17 years. The CHiPs work systematically through their zone, making appointments and repeat visits to try to contact those who are initially absent from home, or who are initially unsure if they wish to participate and/or test for HIV. During R2, new strategies were implemented to try to increase the percentage of men who were contacted by the CHiPs, and to facilitate more rapid linkage to HIV care among HIV-positive individuals. In some zones, CHiPs periodically supplemented their household visits with “zonal campaigns” during which they offered services in a non-household setting, and the frequency with which they worked early and late in the day and on weekends was increased. With respect to linkage to care, there was a greater focus on this in R2 compared with R1, with targetted follow-up of individuals who had been referred but not yet linked to care, increased coordination between the CHiPs and the clinic to facilitate linkage, and clinic improvements.

### Data collection

CHiPs record information on electronic registers, as described elsewhere[18]. Clients verbally consent to participation in the intervention, and provide written consent for HIV counselling and testing. Data captured electronically includes consent to participate in the intervention, self-reported HIV status, the date of the most recent HIV test, uptake of HIV testing, the outcome of HIV testing by CHiPs, and which referrals were given.

Individuals are “known HIV-positive” to the CHiPs in R2 if they participated in R2 and either (i) self-reported or tested HIV-positive in R1, and verbally confirmed their HIV-positive status in R2 (ii) self-reported HIV-positive for the first time in R2, or (iii) tested HIV-positive with CHiPs in R2. For those who confirmed or self-reported they were HIV-positive in R2, self-reported data were collected at the time of the annual R2 visit on whether they were registered for HIV care, had ever taken ART, were currently on ART, and for those who reported taking ART how many pills they had missed in the previous three days. For individuals who reported they were registered for HIV care, CHiPs asked to see the patient ART card; if the card was provided, the ART card number was recorded.

For all who were known HIV-positive following the R2 annual visit, CHiPs were expected to collect updated information at follow-up visits about whether an individual was still resident in the zone, and (if the individual was contacted) self-reported information on registration for HIV care, ART initiation, and ART adherence.

### Outcomes and data analysis

Analyses were done for individuals aged ≥15 years, and separately for men and women. The indicators of intervention coverage used for our analyses were determined at the start of the trial; when the UNAIDS 90-90-90 targets were announced at a later stage during 2014, we extended our indicators to include estimates of coverage against these targets.

Among outcomes that were measured directly among individuals who consented to participate in R2, five are of central interest because they represent the key steps in the HIV testing-and-treatment cascade:

1. the percentage who consented to participate in R2, among all household members who were enumerated;
2. the percentage who knew their HIV status following the R2 annual visit, among all who consented to participate in R2;
3. the percentage who were on ART by the end of R2, among known HIV-positive individuals who remained resident in the same zone of the community at the end of R2;
4. the percentage who were retained on ART on the date of consenting to participate in R2, among known HIV-positive individuals who ever reported to CHiPs (during R1 and/or at the R2 annual visit) that they had ever taken ART;
5. the time to start ART after referral to HIV care by CHiPs in R2, among known HIV-positive individuals who were not on ART on the date of referral.

For knowledge of HIV status following the R2 annual visit, we applied a strict definition: that someone knew their HIV status if they either self-reported HIV-positive, accepted the offer of HIV testing with CHiPs and received their result, or self-reported they had tested HIV-negative in the previous 3 months. We applied a less strict definition for knowledge of HIV status immediately prior to the R2 visit: that someone knew their HIV status if they either self-reported HIV-positive, self-reported they had tested HIV-negative in the previous 12 months, or tested HIV-negative with CHiPs in R1.

We used the Kaplan-Meier “time-to-event” method to estimate the time from first referral in R2 to ART initiation, and Cox regression for analysis of whether the time to ART initiation differed by gender or age group, with censoring on the date of the last follow-up visit at which an individual was contacted in person for those who did not start ART, and using follow-up data up to September 30 2017.

### Estimation of the number of HIV-positive individuals in the population, and the first and second “90s”

Methods for estimating the number of HIV-positive individuals, and the first and second 90s, have been described in detail elsewhere, and have previously been applied to the R1 intervention data from Arm A communities in Zambia[18]. The methods are summarized here.

First, we estimated the number of HIV-positive individuals among all who participated in R2 (those who consented to participate, and also had health counselling information recorded), as the sum of: (1) the number who were known by the CHiPs to be HIV-positive; and (2) an estimated number among those whose HIV status was not known to CHiPs, assuming that HIV prevalence in this group was the same as among those who accepted testing in R2. We extrapolated to the total population who were enumerated in R2 by assuming that HIV prevalence among non-participants in R2 was the same as among participants.

We calculated the first and second 90s, among individuals who participated in R2, as follows:

1. the proportion of HIV-positive individuals who knew their HIV-positive status *immediately before Round 2* = the total who self-reported they were HIV-positive, divided by the estimated number of HIV-positive individuals;
2. the proportion who knew their HIV-positive status *by the end of Round 2* = the total who were known by the CHiPs to be HIV-positive following the R2 annual visit, divided by the estimated number of HIV-positive individuals;
3. the proportion who were on ART *immediately after the Round 2 annual visit*, among those who knew their HIV-positive status = the total who self-reported they were on ART, divided by the number who were known by the CHiPs to be HIV-positive immediately following the R2 annual visit;
4. the proportion who were on ART *by the end of Round 2*, among those who knew their HIV-positive status = the total who self-reported they were on ART at the last CHiP visit made during R2, divided by the number who were known by the CHiPs to be HIV-positive and who remained resident in the same zone of the community according to the last information collected during R2.

We extrapolated to the total population by assuming that (1) knowledge of HIV-positive status and ART uptake *among non-participants* in R2 was the same as among participants *immediately before* the R2 annual visit and (2) these outcomes did not change during R2, equivalent to assuming that non-participants did not receive HIV testing or treatment services during R2.

For R1, all estimation was done with stratification on gender, community of residence, and age group. For R2, we additionally stratified by six categories of prior residency and participation: (1) newly resident in the zone of the community (among whom information was not collected about whether they had moved from within, or from outside, the community); (2) resident in the same zone of the community in R1, but did not participate in R1; (3) participated in R1 and self-reported HIV-positive; (4) participated in R1 and was newly diagnosed HIV-positive; (5) participated in R1 and tested HIV-negative; (6) participated in R1 but did not self-report HIV-positive and did not accept the offer of HIV testing in R1. The stratum-specific estimates of all numbers (for example, the number of HIV-positive individuals, the number of HIV-positive individuals who knew their HIV-positive status) were then summed to provide summaries separately for each age group, according to participation and residency (yes or no) in R1, and overall. We also used sensitivity analysis for key assumptions, as for estimates of progress towards 90–90 after one year of intervention[18].

### Comparison with Arm C (standard-of-care) communities, and findings from Arm A communities in South Africa

We are bound by strict trial oversight procedures that preclude the publication of any comparison across trial arms while the study is ongoing, and because there is no “PopART” intervention in the communities in the control arm there are no CHiP intervention data collected in Arm C.

As explained previously, we did not report estimates of coverage against the 90–90 targets after one year of intervention in South Africa because some challenges were encountered there with data collected as part of the CHiP intervention[18]. Several of these challenges were resolved by the start of year 2, but some new challenges emerged. Following intensive efforts, these challenges were fully resolved by early 2016 but not in time for it to be possible to reliably estimate coverage against the 90–90 targets after two years of intervention.

### Ethical considerations

The study and all the above procedures were approved by the ethics committees of the London School of Hygiene & Tropical Medicine and the University of Zambia. The study is sponsored by NIH.

## Results

### Cascade from enumeration, through participation, to knowledge of HIV status, among all individuals

CHiPs visited 45,631 households during R2, ~98% of the total (as estimated from a census conducted by the study team in 2013) across the four communities. For 94% of visited households, consent was given for the intervention to be explained and for all household members to be listed on the CHiPs’ electronic register, and 120,272 individuals aged ≥15 years were enumerated.

**Fig 1:**
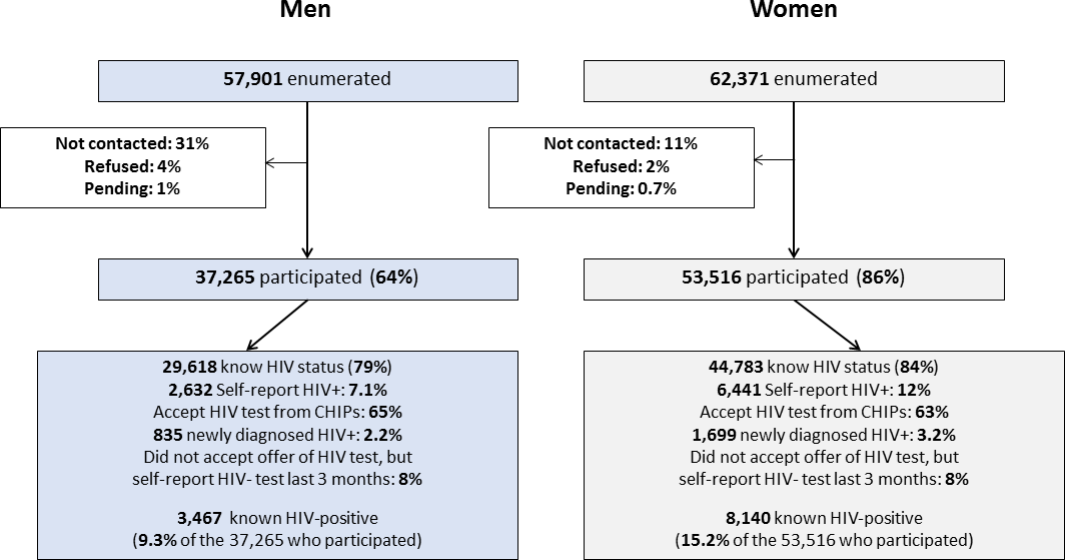
Enumeration, consent to participation, and knowledge of HIV status, in Round 2.

Among enumerated individuals, consent to participate in the PopART intervention was high among women (86%) but lower among men (64%), because it was harder for CHiPs to contact men at home compared with women (Fig 1). Among women, consent to participate was high across all age groups except those aged 15–17 years; among men, consent to participate was highest among those aged 18–24 and ≥60 years (Table 1).

**Table 1:** Cascade from enumeration, through participation and knowledge of HIV status, in Round 2.

	Enumerated	Consented to participate		Known HIV status before Round 2 visit^1^		Known HIV status after Round 2 visit^2^		Self-reported HIV+ in Round 2		Tested with CHiPs in Round 2		Did not test with CHiPs in Round 2, reported HIV-negative test in previous 3 months		Known HIV-positive^3^	
		%^4^	n	%^5^	n	%^5^	n	%^5^	n	%^5^	n	%^5^	n	%^5^	n
Overall	120,272	**75**	90,781	63	57,218	**82**	74,401	**10.0**	9,073	**64**	58,073	**8**	7,255	**12.8**	11,607
Men	57,901	**64**	37,265	57	21,249	**79**	29,618	**7.1**	2,632	**65**	24,177	**8**	2,809	**9.3**	3,467
Women	62,371	**86**	53,516	67	35,789	**84**	44,783	**12.0**	6,441	**63**	33,896	**8**	4,446	**15.2**	8,140
Men, by age															
15–17	3,933	**60**	2,341	17	391	**79**	1,846	0.5	12	75	1,754	3	80	**0.9**	22
18–19	4,725	**76**	3,570	41	1,475	**85**	3,049	0.6	23	79	2,832	5	194	**1.1**	38
20–24	10,391	**72**	7,908	59	4,636	**84**	6,613	0.8	65	76	6,031	7	517	**2.0**	154
25–29	9,257	**64**	5,902	61	3,603	**81**	4,805	3.2	187	69	4,057	10	561	**5.8**	344
30–34	8,079	**59**	4,776	62	2,956	**78**	3,717	8.0	382	61	2,894	9	441	**12.1**	580
35–39	6,666	**58**	3,939	65	2,553	**78**	3,078	14.3	562	54	2,146	9	370	**18.0**	707
40–44	5,126	**58**	2,959	67	1,984	**76**	2,245	19.6	580	48	1,425	8	240	**23.1**	683
45–49	2,984	**58**	1,741	66	1,146	**77**	1,335	20.0	349	50	862	7	124	**24.1**	420
50–54	2,001	**59**	1,183	68	803	**75**	888	19.4	230	48	569	8	89	**21.6**	255
55–59	1,432	**64**	914	67	610	**73**	665	15.1	138	50	458	8	69	**16.5**	151
60–64	976	**70**	679	63	427	**68**	464	8.0	54	53	363	7	47	**8.4**	57
65+	1,791	**76**	1,353	62	845	**67**	913	3.7	50	58	786	6	77	**4.1**	56
Women, by age															
15–17	4,567	**71**	3,265	21	674	**80**	2,620	0.5	16	76	2,470	4	134	**1.6**	52
18–19	5,983	**87**	5,197	53	2,730	**88**	4,556	1.3	66	80	4,182	6	308	**3.4**	175
20–24	14,143	**89**	12,619	69	8,673	**88**	11,069	4.9	622	74	9,302	9	1,145	**8.5**	1,069
25–29	10,230	**90**	9,157	74	6,769	**86**	7,917	11.9	1,090	64	5,823	11	1,004	**16.2**	1,482
30–34	7,834	**88**	6,895	75	5,206	**85**	5,842	18.9	1,302	56	3,887	9	653	**23.4**	1,613
35–39	5,982	**85**	5,085	76	3,842	**84**	4,271	24.6	1,251	50	2,562	9	458	**28.1**	1,428
40–44	4,001	**82**	3,295	76	2,511	**82**	2,709	28.0	921	46	1,526	8	262	**31.0**	1,020
45–49	2,634	**81**	2,129	72	1,543	**79**	1,673	23.0	490	48	1,020	8	163	**25.8**	550
50–54	2,356	**82**	1,936	72	1,402	**78**	1,502	17.2	332	54	1,045	6	125	**19.2**	371
55–59	1,644	**86**	1,407	66	930	**73**	1,023	13.9	195	53	747	6	81	**15.1**	212
60–64	1,130	**86**	967	67	645	**69**	666	10.4	101	53	515	5	50	**10.8**	104
65+	1,867	**84**	1,564	55	864	**60**	935	3.5	55	52	817	4	63	**4.1**	64

1 Counted as knowing HIV status before Round 2 visit if self-reported HIV-positive, reported an HIV-negative test in the previous 12 months, or tested HIV-negative with CHiPs in Round 1

2 Counted as knowing HIV status following Round 2 visit if self-reported HIV-positive, tested with CHiPs in Round 2, or reported an HIV-negative test result in the previous 3 months (stricter definition than (1), to fit with the messaging from CHiPs that if it is >3 months since the last HIV-negative test then it is advised that an individual should accept the offer of testing from CHiPs in order to update their knowledge of their HIV status)

3 “Known HIV-positive” if self-reported or tested HIV-positive in Round 1 and verbally confirmed their HIV-positive status in Round 2, or self-reported HIV-positive for the first time in Round 2, or tested HIV-positive with CHiPs in Round 2

4 Denominator is all enumerated as a household member

5 Denominator is all who consented to participate in Round 2.

Even though we applied a stricter definition for knowledge of HIV status after, compared with before, the R2 annual visit (see Methods), R2 achieved a large increase in the percentage of people who knew their HIV status: from 67% to 84% for women, and from 57% to 79% for men (Table 1). Knowledge of HIV status following the R2 visit was highest among those aged 18–24 years, for both men (84–85%) and women (88%), and then fell with increasing age (Table 1).

The percentage of individuals who self-reported HIV-positive as well as those who were known HIV-positive following the R2 annual visit showed a striking age pattern, with a peak at age 40–49 years for men and at 35–44 years for women (Table 1). Among individuals who had self-reported or tested HIV-positive in R1, and participated again in R2, 98% (5,577/5,700) verbally confirmed their HIV-positive status in R2. Overall, 9.3% of men and 15.2% of women were known HIV-positive, similar to R1[18].

### ART uptake among known HIV-positive individuals at the start and by the end of Round 2

On the date of the R2 annual visit, 64% of known HIV-positive men and 69% of known HIV-positive women self-reported that they were on ART (Table 2). This was substantially higher than at the start of R1 (~50%)[18]. The percentage of individuals on ART showed a striking age pattern, as in R1, increasing from age 20–24 years and stabilizing at ~80% for men ≥50 years and ~80% for women ≥35 years. Correspondingly, the percentage of known HIV-positive individuals who were newly diagnosed HIV-positive by the CHiPs in R2 (22%) was much lower than in R1 (40%), and much higher for younger than older individuals. In terms of absolute numbers, most new HIV-positive diagnoses were among men aged 20–49 years and women aged 18–44 years.

**Table 2:** ART uptake among known HIV-positive individuals who participated in Round 2, at the start and by the end of Round 2.

	Known HIV+ following Round 2 visit	Newly diagnosed HIV+ by CHiPs in Round 2		Self-reported HIV+ at Round 2 annual visit						Remained resident at the end of Round 2		On ART on date of last visit within Round 2, among individuals who remained resident at the end of Round 2	
				At Round 2 visit: Reported never previously registered for HIV care		At Round 2 visit: Reported have previously registered for HIV care, but not currently on ART		At Round 2 visit: Reported currently on ART					
		%^1^	n	%^1^	n	%^1^	n	%^1^	n	%^1^	n	%^2^	n
Overall	11,607	**22**	2,534	6	749	4	519	**67**	7,805	**83**	9,679	**81**	7,801
Men	3,467	**24**	835	6	224	5	179	**64**	2,229	**82**	2,845	**80**	2,269
Women	8,140	**21**	1,699	6	525	4	340	**69**	5,576	**84**	6,834	**81**	5,532
Men, by age													
15–17	22	**45**	10	0	0	0	0	**55**	12	100	22	**77**	17
18–19	38	**39**	15	5	2	11	4	**45**	17	92	35	**66**	23
20–24	154	**58**	89	6	9	4	6	**32**	50	74	114	**52**	59
25–29	344	**46**	157	11	38	5	18	**38**	131	73	252	**60**	152
30–34	580	**34**	198	8	47	5	29	**53**	306	74	430	**77**	332
35–39	707	**21**	145	7	49	6	39	**67**	474	81	573	**81**	467
40–44	683	**15**	103	7	46	6	40	**72**	494	85	581	**82**	478
45–49	420	**17**	71	4	17	5	20	**74**	312	89	375	**85**	317
50–54	255	**10**	25	4	9	4	9	**83**	212	88	225	**92**	207
55–59	151	**9**	13	3	5	5	8	**83**	125	88	133	**91**	121
60–64	57	**5**	3	2	1	2	1	**91**	52	96	55	**96**	53
65+	56	**11**	6	2	1	9	5	**79**	44	89	50	**86**	43
Women, by age													
15–17	52	**69**	36	2	1	0	0	**29**	15	77	40	**52**	21
18–19	175	**62**	109	6	11	2	3	**30**	52	74	129	**60**	78
20–24	1,069	**42**	447	9	100	5	52	**44**	470	73	785	**63**	498
25–29	1,482	**26**	392	8	112	5	67	**61**	911	79	1,172	**75**	877
30–34	1,613	**19**	311	6	104	4	69	**70**	1,129	85	1,374	**82**	1,127
35–39	1,428	**12**	177	5	72	4	54	**79**	1,125	86	1,234	**87**	1,073
40–44	1,020	**10**	99	5	49	4	43	**81**	829	88	901	**89**	804
45–49	550	**11**	60	5	26	5	28	**79**	436	90	496	**88**	435
50–54	371	**11**	39	6	21	3	13	**80**	298	93	344	**86**	297
55–59	212	**8**	17	8	18	3	7	**80**	170	95	201	**87**	174
60–64	104	**3**	3	9	9	3	3	**86**	89	97	101	**95**	96
65+	64	**14**	9	3	2	2	1	**81**	52	89	57	**91**	52

1 The denominator for the percentages is the number of known HIV-positive individuals following the Round 2 visit

2 The denominator is the number of known HIV-positive individuals who remained resident at the end of Round 2 according to the last follow-up visit made by CHiPs in Round 2.

Among those known to be HIV-positive, 82% of men and 84% of women remained resident in the same zone of the community at the end of R2 according to the last CHiP visit in R2 (Table 2), with the lowest figures among men aged 20–34 years and women aged 15–29 years.

By the end of R2, among those who remained resident in the same zone of the community, 80% of known HIV-positive men and 81% of known HIV-positive women were on ART (Table 2). The percentage on ART increased between the start and end of R2 in all age groups, remaining higher among older than younger individuals at the end of R2, and approached or reached 90% for men aged ≥45 years and women aged ≥35 years. An ART card number was recorded for 76% of individuals who reported they were on ART, at the start and/or by the end of R2.

### Retention on ART on the date of first participation in Round 2, among known HIV-positive individuals who had ever taken ART

On the date of first consenting to participate in R2, 2,345 known HIV-positive men and 5,766 known HIV-positive women either reported they had previously taken ART, and/or they had reported prior or current use of ART during R1 (S1 Table). Among this group, 93% of men and 95% of women reported being on ART at the time of the R2 annual visit, and that they had missed 0 pills in the previous 3 days, with figures of >90% for almost all age groups.

### Time from first CHiP referral in Round 2 to ART initiation

**Fig 2:**
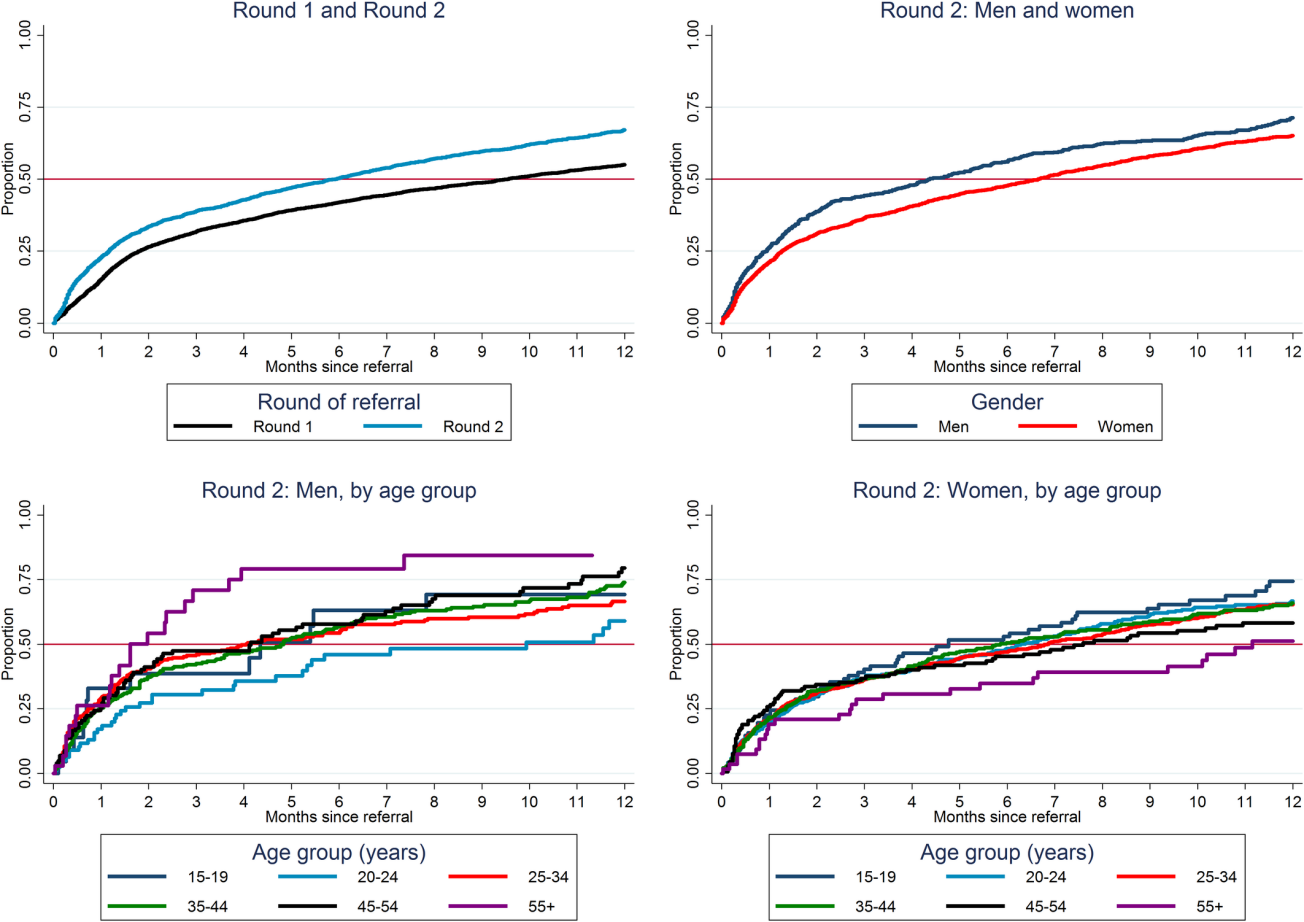
Time to start ART after CHiP referral to HIV care, estimates from “time-to-event” analysis.

Among 3,435 HIV-positive individuals who were not on ART on the date of first participation in R2 and were referred to HIV care, 1,774 were subsequently recorded as linked to HIV care, of whom 95% (1,680/1,774) were recorded as initiated on ART. Overall, we estimated that 23%, 39%, 50%, and 67% started ART by 1, 3, 6, and 12 months respectively after first referral to care in R2 (Fig 2, S2 Table), with a median time to start ART of 6 months. These figures were considerable improvements compared with R1, when an estimated 42% and 55% had started ART by 6 and 12 months respectively after first referral to care with a median time to start ART of 9.5 months (Fig 2). Overall the time to start ART was slightly slower for women than men, among men slowest for those aged 20–24 years, and among women slowest for those aged ≥55 years in R2 (Fig 2, S2 Table).

### Knowledge of HIV-positive status among people living with HIV, among individuals who participated in Round 2

We estimated there were 3,705 HIV-positive men, and 8,515 HIV-positive women, among all who participated in R2 (Table 3), in comparison to 3,467 *known* HIV-positive men and 8,140 *known* HIV-positive women (Table 2). Estimated HIV prevalence was therefore 10.3% in men and 16.3% in women (compared with 9.3% of men and 15.2% of women being *known* HIV-positive).

**Table 3:** 90–90 estimates at the start and end of Rounds 1 and 2, among individuals who participated in the intervention and with extrapolation to the total population.

			First 90		Second 90	
	Estimated number of HIV-positive individuals / total population, %		Immediately before annual round visit	End Round	Immediately following annual round visit	End Round
**Individuals who participated**						
**Men**	n / N^1^	%^2^	%	%	%	%
Round 2, Overall	3,705 / 35,888	10.3	**71**	**94**	**64**	**80**
Round 2, did not participate in Round 1	1,706 / 19,038	9.0	50	91	48	71
Round 2, participated in Round 1	1,999 / 16,850	11.9	89	96	77	86
*Round 1*, *Overall*	*4*,*662 / 45*,*399*	*10*.*3*^4^	*52*	***89***	*47*	***72***
**Women**						
Round 2, Overall	8,515 / 52,210	16.3	**76**	**96**	**69**	**81**
Round 2, did not participate in Round 1	3,667 / 25,764	14.2	57	93	55	73
Round 2, participated in Round 1	4,848 / 26,446	18.3	89	97	78	86
*Round 1*, *Overall*	*9*,*499 / 55*,*703*	*17*.*1*^4^	*56*	***92***	*49*	***72***
**Extrapolation to total population**						
**Men**	n / N^3^	%^2^	%	%	%	%
Round 2, Overall	6,521 / 61,332	10.6	**67**	**79**	**71**	**81**
Round 2, did not participate in Round 1	3,665 / 36,002	10.2	51	70	63	77
Round 2, participated in Round 1	2,856 / 25,330	11.3	87	92	78	85
*Round 1*, *Overall*	*6*,*649 / 61*,*606*	*10*.*8*^4^	*52*	***78***	*54*	***74***
**Women**						
Round 2, Overall	10,690 / 66,106	16.2	**75**	**91**	**71**	**82**
Round 2, did not participate in Round 1	4,805 / 33,910	14.2	58	86	61	76
Round 2, participated in Round 1	5,885 / 32,196	18.3	89	96	79	86
*Round 1*, *Overall*	*11*,*037 / 64*,*305*	*17*.*2*^4^	*56*	***87***	*53*	***73***
**Men and Women**						
Round 2, Overall	17,211 / 127,438	13.5	72	**87**	71	**82**
*Round 1*, *Overall*	*17*,*686 / 125*,*911*	*14*.*0*	*55*	***83***	*53*	***73***

1 n = estimated number of HIV+ individuals among all who participated in the round, N = total who participated in the round = total who consented to participate in the round AND had health counselling information recorded

2 estimated HIV prevalence

3 n = estimated number of HIV+ individuals among total population, N = total estimated population

4 Round 1 estimates are restricted to individuals aged ≥18 years on the date they were first enumerated in Round 1, whereas Round 2 estimates include those aged 15–17 years as well as those aged ≥18 years on the date they were first enumerated in Round 2.

**Fig 3:**
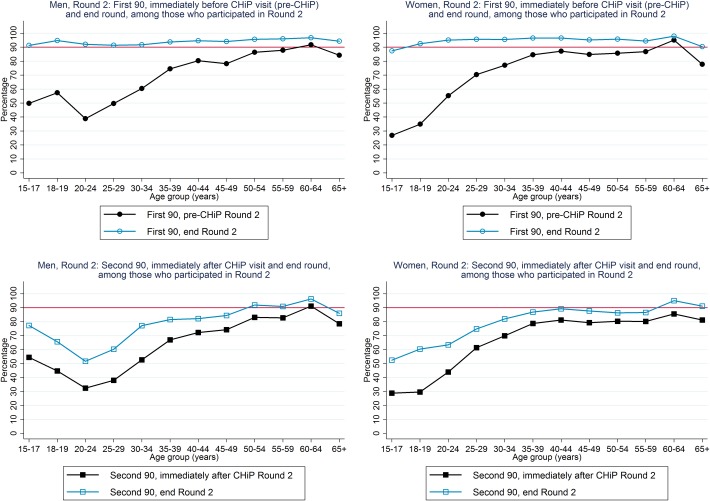
First and second 90 estimates, among individuals who participated in Round 2.

An estimated 71% of HIV-positive men and 76% of HIV-positive women knew their HIV-positive status immediately before the R2 annual visit (“pre-CHiP”), higher for older than younger individuals, and increasing to ~95% for both men and women by the end of R2 (Table 3, Fig 3).

Among both HIV-positive men and HIV-positive women, pre-CHiP knowledge of HIV-positive status was very different in those who did, and did not, participate in R1 (in the CHiP zone in which they were resident in R2). For example, among HIV-positive men who did not participate in R1 we estimated that immediately before the R2 annual visit 50% knew their HIV-positive status; the corresponding estimate among those who participated in R1 was 89% (Table 3).

### Population-level estimates of the first and second 90s, and ART coverage, among all HIV-positive individuals irrespective of whether they participated in Round 2

For estimation of the first and second 90s among the *total* population of people living with HIV, assumptions are required for individuals who did not participate in R2 (see Methods).

We estimated there were 6,521 HIV-positive men and 10,690 HIV-positive women in the total population, among whom 57% (n = 3,705) of HIV-positive men and 80% (n = 8,515) of HIV-positive women participated in R2 (Table 3). Estimated HIV prevalence was 10.6% in men and 16.2% in women.

**Fig 4:**
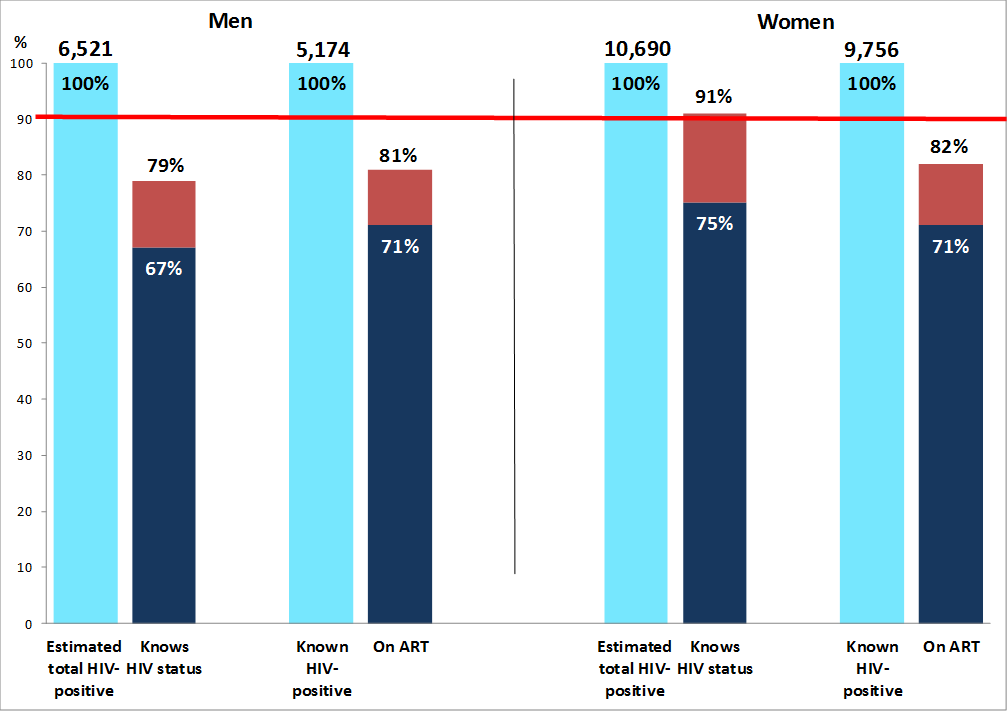
90–90 estimates for the total population, at the start and end of Round 2. Dark blue bars show the estimated percentage of HIV+ individuals who knew their HIV+ status (first 90 target) and the estimated percentage who were on ART among those who knew their HIV+ status (second 90 target), immediately prior to the Round 2 annual visit. Red bars show the same estimated percentages, by the end of Round 2. ART, antiretroviral therapy.

**Fig 5:**
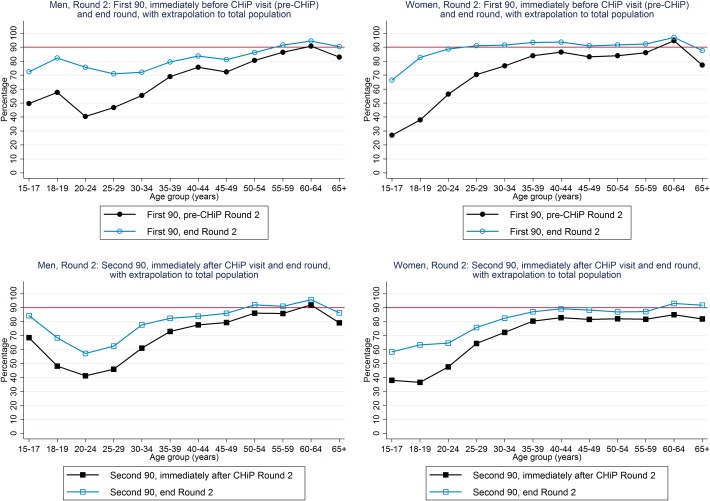
First and second 90 estimates, with extrapolation to total population in Round 2.

For the “first 90”, we estimated that 67% of the total population of HIV-positive men and 75% of the total population of HIV-positive women knew their HIV-positive status immediately before the R2 annual visit, increasing to 79% and 91% by the end of R2 (Table 3 and Fig 4). Among HIV-positive men, knowledge of HIV-positive status by the end of R2 was ~70–80% for those aged 15–49 years and ~90% among those ≥50 years. Among HIV-positive women, knowledge of HIV-positive status immediately before the R2 annual visit approached 90% among those aged ≥35 years, and by the end of R2 was ~90% among those aged ≥20 years (Fig 5).

For the “second 90”, we estimated that 71% of known HIV-positive men and 71% of known HIV-positive women were on ART immediately following the R2 annual visit, increasing to 81% and 82% respectively by the end of R2 (Table 3 and Fig 4). Among known HIV-positive men, the percentage on ART by the end of R2 was >80% among those aged ≥35 years but much lower at ~60% among those aged 20–29 years. Among known HIV-positive women, the percentage on ART by the end of R2 was approaching 90% for those aged ≥35 years and was in the range 60–80% for younger women (Fig 5).

**Fig 6:**
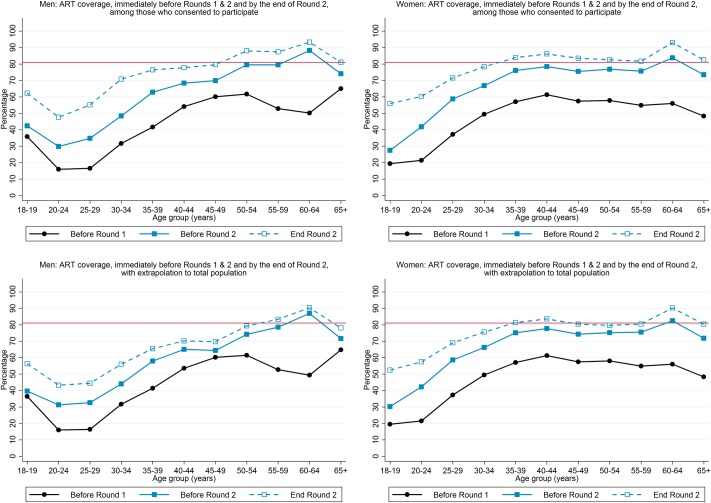
ART coverage, immediately before Rounds 1 and 2, and by the end of Round 2.

We estimated that ART coverage was 65% among HIV-positive men and 75% among HIV-positive women by the end of R2, compared with the cumulative 90–90 target of 81%, and close to 81% for HIV-positive men aged ≥50 years and HIV-positive women aged ≥35 years but lower for younger individuals (Fig 6). It was striking that ART coverage immediately before the R2 annual visit was substantially higher than immediately before the R1 annual visit, for both men and women, across the age range (Fig 6).

Among both men and women, population-level pre-CHiP knowledge of HIV-positive status was very different in those who did, and did not, participate in R1 (in the CHiP zone in which they were resident in R2). For example, among HIV-positive men who did not participate in R1 we estimated that immediately before the R2 annual visit 51% knew their HIV-positive status; the corresponding estimate among those who participated in R1 was 87%. The disparity remained at the end of R2, with figures of 70% and 92% respectively (Table 3). There was also a disparity between those who did, and did not, participate in R1 for the second 90; for example, among known HIV-positive men who did not participate in R1 we estimated that 77% were on ART by the end of R2, compared with 85% among those who participated in R1 (Table 3). Among individuals who did not participate in R1, the percentage who knew their HIV-positive status immediately before the R2 visit was lower, but increased more by the end of R2, among those who were *not resident* in the same zone in R1 compared with those who were *resident in the same zone in R1 but did not participate* (S3 Table); this pattern was also seen for the percentage on ART among those who knew their HIV-positive status.

The disparities between those who did, and did not participate, in R1 are important in the context that among the estimated total of HIV-positive men and women, ~55% and ~45% respectively did not participate (in the CHiP zone in which they were resident in R2) in the intervention in R1 (Table 3), and ~40% were not resident (in the CHiP zone in which they were resident in R2) in R1.

## Discussion

### Summary of findings

After two years of delivering the PopART UTT intervention in four peri-urban/urban communities in Zambia, with changes in national ART guidelines and programming occurring in parallel, substantial progress was made towards reaching 90–90. We estimated that the first 90 was reached among HIV-positive women, that ~80% of HIV-positive men knew their HIV-positive status, that ~80% were on ART among HIV-positive men and women who knew their HIV-positive status, and that ART coverage was ~65% among HIV-positive men and ~75% among HIV-positive women compared with the cumulative 90–90 target of 81%. For the first 90, the figure for men was similar to, and the figure for women slightly higher than, our estimates after one year of the intervention, and for the second 90 and ART coverage they were ~10% higher. The median time to ART initiation after referral to care by CHiPs was nearly halved in year 2 of the intervention, compared with year 1, among both men and women. The third 90 may have been reached among HIV-positive individuals who had taken ART, based on self-reported information on treatment adherence, but with the caveat that self-reported information can provide an over-estimate of treatment adherence compared with biological testing for ART drugs[19] and that treatment adherence is not equivalent to viral suppression.

At the same time, our findings indicate that reaching 90–90 is hard. The PopART intervention is intensive, with each CHIP team responsible for covering a population of ~500 households over a one-year period, and offering a range of combination HIV prevention services in addition to universal testing and support for linkage to HIV care. Two years have not been enough to achieve 90–90.

### Comparison with other studies

Our results can be compared with those from the SEARCH CRT, a trial which aims to reduce HIV incidence through a combination HIV prevention intervention that includes UTT in 16 intervention communities in rural Uganda and Kenya. Each community has a population of ~10,000 people, and it has been reported that among stable residents (defined as those who, at the time of first enumeration of the study population, had been resident in the community for ≥6 of the previous 12 months), 90-90-90 was achieved after two years of intervention[20]. Trial interventions include community campaigns, mobile HIV testing in combination with multi-disease screening followed by a targeted offer of home-based HIV testing, and various strategies to support linkage to care and ART initiation and adherence[13, 21]. In the BCPP CRT, 90-90-90 was close to being achieved at baseline, and very rapid ART initiation has been achieved among individuals who were not on ART at the time of first participation in the intervention[22]. In contrast, ART coverage in the UTT intervention communities of the TasP study in rural South Africa was far below the cumulative 90–90 target after two years of intervention[23], and much lower than what has been achieved after two years in the PopART Arm A communities in Zambia.

### Explanations for our findings, and remaining gaps and how to close them

The main reason why the first 90 has not yet been reached among men is because of difficulties in contacting them, despite CHiPs regularly working on weekends, early and late in the day, periodically offering services outside the household at times and places that are anticipated to be convenient, and offering HIV testing as part of a wider range of services, all of which facilitate testing uptake[24–26]. Nevertheless, ~75% of men in the year 2 population participated in at least one of years 1 and 2.

Progress towards the second 90 has been slower than towards the first 90. Nonetheless, ~70% of known HIV-positive individuals were on ART at the start of year 2, compared with ~50% at the start of year 1, and during year 2 there was an increase across all age groups to reach the overall figure of ~80% by the end of year 2. The shorter time to initiate ART following referral by CHiPs in year 2, compared with year 1, was likely attributable to multiple factors. These included the increased focus of the CHiPs on linkage to care in year 2, compared with year 1, together with targetted follow-up of individuals who had been referred but not yet linked to care; fewer newly diagnosed HIV-positive individuals in year 2 compared with year 1; increasing community acceptance and understanding of the CHiPs, and of ART and UTT, with time; increased coordination between the CHiPs and the clinic to facilitate linkage to care; and clinic improvements. It was not possible to implement a “same-day” ART initiation (on the same day that an individual first links to HIV care) policy but despite this 95% of those who were known to have linked to care were also known to have initiated ART. For comparison with our findings, in the BCPP CRT a high percentage of individuals linked to HIV care within a short time after referral, facilitated by SMS reminders, incentives following linkage to care, and follow-up visits to those who did not attend their first clinic appointment; and in the SEARCH trial a high percentage linked to HIV care by 12 months after referral, facilitated by streamlined care and a patient-centred approach to care[21, 22].

The main ART coverage gap that remained to be closed after two years of intervention was among men aged <50 years, women aged <35 years, and individuals who did not participate in year 1. An important finding from year 2 was the high proportion (~50%) of individuals who had not participated in year 1, about 80% of whom were newly resident in the area of the community (CHiP zone) in which they were living at the time of the year 2 visit. Among this group, we estimated that only ~50% of HIV-positive individuals knew their HIV-positive status prior to year 2, similar to our finding across the total population prior to year 1. This “challenge of mobility” has also been described in the study population of the TasP trial[27]. This population movement has slowed, but not reversed, progress towards 90–90 in the PopART study communities.

In year 1 of the intervention ~40% of known HIV-positive individuals were newly diagnosed by the CHiPs. Despite the challenge of mobility, and that ~15% of the year 1 population did not participate in the intervention in year 1, this figure fell to ~20% in year 2. That the figure was as high as ~20% shows the value of the second year of intervention for diagnosing new HIV infections, in particular for individuals who had not participated in year 1 but also through repeat testing of those who tested HIV-negative in year 1 and those who declined the offer of HIV testing in year 1 but accepted a test in year 2.

### Study strengths and limitations

A limitation has been the relatively high percentage of men whom the CHiPs have not contacted, which limits intervention effectiveness and means that for ~one-third of the male population assumptions have been required to estimate testing and treatment coverage in the total population. In sensitivity analyses in which the values of HIV prevalence and testing and treatment coverage were varied among participants whose HIV status was not known to CHiPs, the range of values for coverage against the first and second 90 targets was from ~3% lower to ~2% higher than our central estimates (S4 Table). A strength has been that CHiPs attempted to reach the entire resident adult population in year 2 of the intervention, irrespective of whether a household or individual was resident in year 1.

Information on uptake of treatment, and treatment adherence, has relied on self-reported information; linkage to clinic records has not been possible. However, by year 2 of the intervention CHiPs had in general established good relationships and trust with their clients, and they have received thorough and ongoing training on electronic data collection, giving overall confidence in the self-reported data. Also, our estimates of HIV prevalence are compatible with previous research data[28], and baseline findings among individuals enrolled into the research cohort study through which the trial primary endpoint will be measured.

Our measure of treatment adherence is a simple one, calculated as a “snapshot” measure on the date of the annual round 2 visit, but with the advantage that the “third 90” is conceptually also a “snapshot” measure. The CHiP database, in which all the information recorded by CHiPs is held, does not allow an individual’s records to be linked if they move from one CHiP zone to another, limiting which individuals can be included in a “cohort analysis” approach to measuring retention on ART at fixed time points after ART initiation. In addition, CHiP follow-up visits are made irregularly, not according to a schedule, and CHiPs may try to follow-up clients but without success.

During year 1 of the intervention, ~38,000 randomly-selected adults were enrolled into a cohort study across all study communities, to measure the primary endpoint of the trial. For all individuals who were HIV-positive at enrolment (based on laboratory testing of a venous blood sample) and were followed up during mid-2016 to mid-2017, their viral load has been measured. These data will provide an estimate of the percentage of HIV-positive individuals who were virally suppressed after two-three years of intervention, and a comparison with the communities that were randomised to standard-of-care.

## Conclusion

Overall coverage against the 90–90 targets was high after two years of the PopART UTT intervention, but was lower among men, young adults, those who were resident in year 1 but participated for the first time in year 2, and those who were newly resident in the area of the community in which they were living in year 2. The median time to ART initiation after referral to care in year 2 was nearly halved compared with year 1, attributable to multiple factors. The third year of intervention spans October 2016 to ~September 2017, and is expected to achieve further progress, especially towards the second 90.

## Supporting information

S1 Table Self-reported retention on ART on the date of first participation in Round 2, among individuals who ever reported (in Round 1 and/or Round 2) to CHiPs that they have ever taken ART. (DOCX)

S2 Table Time to initiate ART after first CHiP referral to HIV care in Round 2. (DOCX)

S3 Table 90–90 estimates at the start and end of Round 2, among individuals who participated in the intervention and with extrapolation to the total population, stratified on their participation and residency (in the same CHiP zone) in Round 1 (R1). (DOCX)

S4 Table Sensitivity analysis of 90–90 estimates in Round 2. (DOCX)

S1 Data Aggregate dataset. (XLSX)

S2 Data Definition of variables in aggregate dataset. (DOCX)
