# Supplementary material for: Towards 90-90: Findings after two years of the HPTN 071 (PopART) cluster-randomized trial of a universal testing-and-treatment intervention in Zambia
Source: PLoS One. 2018 Aug 10;13(8):e0197904. doi: 10.1371/journal.pone.0197904 (PMC6086421; doi:10.1371/journal.pone.0197904)
Supplement: S1 Table — (DOCX) [file pone.0197904.s001.docx]

**S1 Table: Self-reported retention on ART on the date of first participation in Round 2, among individuals who ever reported (in Round 1 and/or Round 2) to CHiPs that they have ever taken ART**

|  |  | All who have ever reported current or previous use of ART |  | All who have ever reported current or previous use of ART, and reported they first started ART before 2014 |  | All who have ever reported current or previous use of ART, and reported they first started ART during 2014-2016 |
| --- | --- | --- | --- | --- | --- | --- |
|  | %^1^ | n/N^2^ | %^1^ | n/N^2^ | %^1^ | n/N^2^ |
| Overall | **95** | 7,680 / 8,111 | **95** | 4,534 / 4,762 | **94** | 3,146 / 3,349 |
| Men | **93** | 2,187 / 2,345 | **94** | 1,291 / 1,378 | **93** | 896 / 967 |
| Women | **95** | 5,493 / 5,766 | **96** | 3,243 / 3,384 | **94** | 2,250 / 2,382 |
| Men, by age |  |  |  |  |  |  |
| 15-17 | **100** | 12 / 12 | **100** | 7 / 7 | **100** | 5 / 5 |
| 18-19 | **84** | 16 / 19 | **81** | 13 / 16 | **100** | 3 / 3 |
| 20-24 | **91** | 48 / 53 | **94** | 15 / 16 | **89** | 33 / 37 |
| 25-29 | **89** | 127 / 142 | **86** | 42 / 49 | **91** | 85 / 93 |
| 30-34 | **93** | 301 / 322 | **90** | 122 / 135 | **96** | 179 / 187 |
| 35-39 | **94** | 465 / 496 | **94** | 237 / 251 | **93** | 228 / 245 |
| 40-44 | **93** | 487 / 525 | **95** | 317 / 335 | **89** | 170 / 190 |
| 45-49 | **93** | 304 / 326 | **94** | 218 / 232 | **91** | 86 / 94 |
| 50-54 | **95** | 209 / 219 | **96** | 161 / 167 | **92** | 48 / 52 |
| 55-59 | **94** | 123 / 131 | **94** | 88 / 94 | **95** | 35 / 37 |
| 60-64 | **98** | 52 / 53 | **97** | 32 / 33 | **100** | 20 / 20 |
| 65+ | **91** | 43 / 47 | **91** | 39 / 43 | **100** | 4 / 4 |
| Women, by age |  |  |  |  |  |  |
| 15-17 | **100** | 15 / 15 | **100** | 9 / 9 | **100** | 6 / 6 |
| 18-19 | **93** | 50 / 54 | **91** | 21 / 23 | **94** | 29 / 31 |
| 20-24 | **92** | 460 / 500 | **94** | 126 / 134 | **91** | 334 / 366 |
| 25-29 | **95** | 894 / 942 | **95** | 374 / 394 | **95** | 520 / 548 |
| 30-34 | **95** | 1,107 / 1,167 | **95** | 628 / 658 | **94** | 479 / 509 |
| 35-39 | **96** | 1,110 / 1,152 | **96** | 744 / 772 | **96** | 366 / 380 |
| 40-44 | **96** | 820 / 853 | **96** | 591 / 614 | **96** | 229 / 239 |
| 45-49 | **95** | 431 / 455 | **96** | 307 / 321 | **93** | 124 / 134 |
| 50-54 | **95** | 295 / 310 | **96** | 222 / 232 | **94** | 73 / 78 |
| 55-59 | **98** | 170 / 174 | **98** | 119 / 122 | **98** | 51 / 52 |
| 60-64 | **98** | 89 / 91 | **97** | 65 / 67 | **100** | 24 / 24 |
| 65+ | **98** | 52 / 53 | **97** | 37 / 38 | **100** | 15 / 15 |

1 % retained on ART on the date of first participation in Round 2. Counted as retained on ART if reported currently taking ART and taking all pills in the previous 3 days, on the date of the annual Round 2 visit; 2 n= retained on ART, N= total who have ever reported to CHiPs that they have ever taken ART
