# Supplementary material for: Towards 90-90: Findings after two years of the HPTN 071 (PopART) cluster-randomized trial of a universal testing-and-treatment intervention in Zambia
Source: PLoS One. 2018 Aug 10;13(8):e0197904. doi: 10.1371/journal.pone.0197904 (PMC6086421; doi:10.1371/journal.pone.0197904)
Supplement: S2 Table — (DOCX) [file pone.0197904.s002.docx]

**S2 Table: Time to initiate ART after first CHiP referral to HIV care in Round 2**

|  | **Number referred to HIV care** | **ART initiated (%)^1^** | | | | **Hazard ratio, unadjusted** | **Hazard ratio, adjusted^2^** | **95% CI** |
| --- | --- | --- | --- | --- | --- | --- | --- | --- |
|  |  | **1 month** | **3 months** | **6 months** | **12 months** |  |  |  |
| **Overall** | 3,435 | 23  95%CI 21-24  *(n=2,570)^3^* | 39  95%CI 37-41  *(n=2,347)* | **50**  95%CI 48-52  *(n=2,188)* | **67**  95%CI 65-69  *(n=1,973)* |  |  |  |
| **Gender** |  |  |  |  |  |  |  |  |
| Men | 1,117 | 26 | 44 | **56** | **71** | 1 (ref) | **1 (ref)** | *P<0.001^5^* |
| Women | 2,318 | 21 | 37 | **48** | **65** | 0.82 | **0.82** | **0.73-0.91** |
| **Men, Age group (years)** |  |  |  |  |  |  |  |  |
| 15-19 | 29 | 33 | 39 | **63** | **69** | 1.01 | **0.95** | **0.54-1.67** |
| 20-24 | 100 | 17 | 31 | **46** | **59** | 0.70 | **0.72** | **0.50-1.03** |
| 25-34 | 453 | 29 | 46 | **54** | **67** | 1 (ref) | **1 (ref)** | *P=0.04* |
| 35-44 | 369 | 25 | 43 | **57** | **74** | 1.03 | **1.06** | **0.86-1.31** |
| 45-54 | 132 | 25 | 47 | **58** | **79** | 1.17 | **1.18** | **0.90-1.55** |
| 55+ | 34 | 26 | 71 | **79** | **/^4^** | 1.71 | **1.75** | **1.11-2.75** |
| **Women, Age group (years)** |  |  |  |  |  |  |  |  |
| 15-19 | 152 | 23 | 40 | **53** | **74** | 1.17 | **1.10** | **0.84-1.43** |
| 20-24 | 556 | 19 | 37 | **48** | **67** | 1.03 | **1.03** | **0.88-1.21** |
| 25-34 | 968 | 21 | 36 | **47** | **65** | 1 (ref) | **1 (ref)** | *P=0.24* |
| 35-44 | 428 | 22 | 37 | **51** | **66** | 1.03 | **1.04** | **0.88-1.23** |
| 45-54 | 154 | 26 | 37 | **45** | **58** | 0.90 | **0.89** | **0.70-1.15** |
| 55+ | 60 | 19 | 29 | **35** | **51** | 0.67 | **0.66** | **0.44-0.99** |

1 Estimated from “time to event” analysis; 2 For overall comparison of women with men, adjusted hazard ratios are obtained from a multivariable Cox regression model including community, age group, and gender; for comparison across age groups, adjusted hazard ratios are obtained from gender-specific multivariable Cox regression models including community and age group; age-specific estimates are presented separately for men and women because there was statistical evidence the age pattern was different for men and women (p=0.004); 3 Number who either started ART within 1 month after referral or have a follow-up visit ≥1 month after referral, and similarly for other time points (3, 6, 12 months after referral); 4 / = Cannot be estimated, because no one followed up to this time point; 5 P-values are from Cox regression, from likelihood ratio tests of whether there is evidence of association between an individual characteristic (e.g. gender) and the outcome of “time to ART initiation”.
