# Supplementary material for: Towards 90-90: Findings after two years of the HPTN 071 (PopART) cluster-randomized trial of a universal testing-and-treatment intervention in Zambia
Source: PLoS One. 2018 Aug 10;13(8):e0197904. doi: 10.1371/journal.pone.0197904 (PMC6086421; doi:10.1371/journal.pone.0197904)
Supplement: S3 Table — (DOCX) [file pone.0197904.s003.docx]

**S3 Table: 90-90 estimates at the start and end of Round 2, among individuals who participated in the intervention and with extrapolation to the total population, stratified on their participation and residency (in the same CHiP zone) in Round 1 (R1)**

|  |  | | First 90 | | Second 90 | |
| --- | --- | --- | --- | --- | --- | --- |
|  | Estimated number of HIV-positive individuals / total population, % | | Immediately before annual round visit | End Round | Immediately following annual round visit | End Round |
| **Individuals who participated** |  | |  |  |  |  |
| **Men** | n / N^1^ | %^2^ | % | % | % | % |
| Overall | 3,705 / 35,888 | 10.3 | 71 | 94 | 64 | 80 |
| Resident in the same CHiP zone in R1, aged ≥18 years at time of R1, but did not participate in R1 | 258 / 2,624 | 9.8 | **54** | **89** | **51** | **69** |
| Not resident (in the CHIP zone in which they were resident in R2) in R1^3^, or aged <18 years at time of R1 | 1,448 / 16,414 | 8.8 | **50** | **91** | **47** | **72** |
| Participated in R1 | 1,999 / 16,850 | 11.9 | 89 | 96 | 77 | 86 |
| **Women** |  | |  |  |  |  |
| Overall | 8,515 / 52,210 | 16.3 | 76 | 96 | 69 | 81 |
| Resident in the same CHiP zone in R1, aged ≥18 years at time of R1, but did not participate in R1 | 252 / 1,741 | 14.5 | **70** | **92** | **64** | **78** |
| Not resident (in the CHIP zone in which they were resident in R2) in R1, or aged <18 years at time of R1 | 3,415 / 24,023 | 14.2 | **57** | **93** | **54** | **73** |
| Participated in R1 | 4,848 / 26,446 | 18.3 | 89 | 97 | 78 | 86 |
|  |  |  |  |  |  |  |
| **Extrapolation to total population** |  |  |  |  |  |  |
| **Men** | n / N^4^ | %^2^ | % | % | % | % |
| Overall | 6,521 / 61,332 | 10.6 | 67 | 79 | 71 | 81 |
| Resident in the same CHiP zone in R1, aged ≥18 years at time of R1, but did not participate in R1 | 826 / 7,512 | 11.0 | **53** | **64** | **70** | **78** |
| Not resident (in the CHIP zone in which they were resident in R2) in R1, or aged <18 years at time of R1 | 2,839 / 28,490 | 10.0 | **50** | **71** | **61** | **77** |
| Participated in R1 | 2,856 / 25,330 | 11.3 | 87 | 92 | 78 | 85 |
| **Women** |  | |  |  |  |  |
| Overall | 10,690 / 66,106 | 16.2 | 75 | 91 | 71 | 82 |
| Resident in the same CHiP zone in R1, aged ≥18 years at time of R1, but did not participate in R1 | 534 / 3,615 | 14.8 | **69** | **79** | **74** | **81** |
| Not resident (in the CHIP zone in which they were resident in R2) in R1, or aged <18 years at time of R1 | 4,271 / 30,295 | 14.1 | **57** | **87** | **59** | **75** |
| Participated in R1 | 5,885 / 32,196 | 18.3 | 89 | 96 | 79 | 86 |

1 n = estimated number of HIV+ individuals among all who participated in the round, N = total who participated in the round; 2 estimated HIV prevalence; 3 **For individuals who were not resident (in the CHiP zone in which they were resident in Round 2) in R1 (a) it is not known whether they moved into the R2 zone from *outside* the community, or from *within* the community (from a different zone), and (b) it is not known whether or not they *participated* in year 1 of the intervention in a different zone**; 4 n = estimated number of HIV+ individuals among total population, N = total estimated population
