# Supplementary material for: Towards 90-90: Findings after two years of the HPTN 071 (PopART) cluster-randomized trial of a universal testing-and-treatment intervention in Zambia
Source: PLoS One. 2018 Aug 10;13(8):e0197904. doi: 10.1371/journal.pone.0197904 (PMC6086421; doi:10.1371/journal.pone.0197904)
Supplement: S4 Table — (DOCX) [file pone.0197904.s004.docx]

**S4 Table: Sensitivity analysis of 90-90 estimates in Round 2**

|  | **Men** | | | | | **Women** | | | | |
| --- | --- | --- | --- | --- | --- | --- | --- | --- | --- | --- |
|  |  | **First 90 (%)** | | **Second 90 (%)** | |  | **First 90 (%)** | | **Second 90 (%)** | |
|  |  | Immediately before annual round 2 visit | End round | Immediately after annual round 2 visit | End round |  | Immediately before annual round 2 visit | End round | Immediately after annual round 2 visit | End round |
|  | N^1^ |  |  |  |  | N^1^ |  |  |  |  |
| Participated in Round 2 | 3,705 | 71 | 94 | 64 | 80 | 8,515 | 76 | 96 | 69 | 81 |
| *Sensitivity analysis^2^* | *3,942* | *70-73* | *91-94* | *63-65* | *77-80* | *8,891* | *75-77* | *94-96* | *67-69* | *79-81* |
| Extrapolated to total population | 6,521 | 67 | 79 | 71 | 81 | 10,690 | 75 | 91 | 71 | 82 |
| *Sensitivity analysis^2^* | *7,016* | *65-69* | *77-81* | *68-72* | *78-82* | *11,181* | *74-77* | *90-92* | *70-72* | *80-82* |

^1^ Estimated number of HIV-positive adults in the population, with extrapolation for (1) individuals who participated but whose HIV status is not known to the CHiPs (2) adults who did not participate

2 Sensitivity analysis: Among adults who participated but their HIV status is not known to CHiPs, HIV prevalence is assumed to be twice the value observed among adults who accepted the offer of HIV testing from CHiPs. Among adults whose HIV status is not known to CHiPs, and they are HIV-positive, two alternative assumptions are made for the percentage who know their HIV+ status (a) the percentage who know their HIV+ status is the *same* as the “pre-CHiP” value for adults whose HIV status is known to CHiPs i.e. equal to (self-reported HIV+ to CHiPs / total known by the CHiPs to be HIV+) (b) the percentage who know their HIV+ status is *half* of the pre-CHiP value among adults whose HIV status is known to CHiPs. Among adults who know their HIV+ status but do not disclose this to CHiPs, two alternative assumptions are made for the percentage who were on ART at the time of the annual round visit (a) the percentage on ART is the same as for adults who self-reported to CHiPs that they were HIV+ (b) the percentage on ART is *half* of the value among adults who self-reported to CHiPs that they were HIV+.
