# Supplementary material for: Towards 90-90: Findings after two years of the HPTN 071 (PopART) cluster-randomized trial of a universal testing-and-treatment intervention in Zambia
Source: PLoS One. 2018 Aug 10;13(8):e0197904. doi: 10.1371/journal.pone.0197904 (PMC6086421; doi:10.1371/journal.pone.0197904)
Supplement: S2 Data — (DOCX) [file pone.0197904.s006.docx]

**Definition of variables on aggregated dataset**

| Variable name | Variable description | Coding |
| --- | --- | --- |
| gender | Gender | 1= Male; 2=Female |
| community | Community of residence | 1,2,3,4 |
| agegroup | Age group (years) | 0=15-17; 1=18-19; 2=20-24; 3=25-29 4=30-34; 5=35-39; 6=40-44; 7=45-49; 8=50-54; 9=55-59; 10=60-64; 11=65+ |
| rd2_residents | Participation and residency in Round 1 (R1) | 0=Resident in R1, did not consent to participate;  1=Participated in R1, self-reported HIV-positive;  2=Participated in R1, tested HIV-positive;  3=Participated in R1, tested HIV-negative;  4=Participated in R1, but did not self-report HIV-positive and did not accept the offer of HIV testing;  5=Newly resident in the CHiP zone in which they were resident at the start of Round 2, and/or aged <18 years at time of Round 1 |
| prop_hh_enumerated | Proportion of households that consented to enumeration, among visited households |  |
| enumerated | Enumerated as a household member | Count of individuals |
| consent_participate | Consented to participate in intervention | Count of individuals |
| participated | Participated in Round 2 (health counselling was done, following consent to participate) | Count of individuals |
| know_hivstatus_pre_r2 | Knew HIV status on date of first participation in R2 (self-reported HIV-positive, or reported their last HIV test was within the previous 12 months and the result was HIV-negative, or they tested HIV-negative with CHiPs in R1) | Count of individuals |
| hivpos_nd | Tested HIV-positive at R2 annual visit | Count of individuals |
| hivpos_srpos_never_reg | Self-reported HIV-positive at R2 annual visit, and reported never previously registered for HIV care | Count of individuals |
| hivpos_srpos_ever_reg_not_art | Self-reported HIV-positive at R2 annual visit, reported previously registered for HIV care, not currently on ART | Count of individuals |
| self_report_hivpos | Self-reported HIV-positive at R2 annual visit | Count of individuals |
| tested_for_hiv | Tested for HIV at R2 annual visit, among those who did not self-report HIV-positive | Count of individuals |
| tested_hiv_negative_3M | Did not self-report HIV-positive, and did not accept offer of HIV testing, but self-reported an HIV-negative test result in the 3 months prior to the Round 2 annual visit | Count of individuals |
| know_hivstatus | Knows HIV status immediately after Round 2 annual visit (defined as self-reported HIV-positive, tested with CHiPs in Round 2, or as part of the R2 annual visit they reported an HIV-negative test result in the previous 3 months) | Count of individuals |
| tested_hivpos | Tested HIV-positive in Round 2 | Count of individuals |
| known_hivpos | Known HIV-positive (self-reported HIV-positive plus tested HIV-positive) following Round 2 visit | Count of individuals |
| on_art_baseline | Self-reported on ART at time of Round 2 annual visit | Count of individuals |
| known_hivpos_resident_end_round2 | Resident in the same area of the community at the end of Round 2 according to last information collected in Round 2, among individuals who were known to be HIV-positive | Count of individuals |
| on_art_end_round2 | On ART at the end of Round 2, among those still resident in the same area of the community according to the last information collected in Round 2 | Count of individuals |
